# Automated opportunistic screening for low bone mineral density using routine CT brain imaging

**DOI:** 10.3389/fradi.2026.1901958

**Published:** 2026-08-14

**Authors:** Rory Zhang, Nishant Panchal, Heinrik Choong, Charlie Ding, Haotian Huang, Stefan Kachel, Cherie Chiang, Numan Kutaiba, Ruth P. Lim

**Affiliations:** 1 Melbourne Bioinnovation Student Initiative (MBSI), Parkville, VIC, Australia; 2 Artificial Intelligence in Radiology Laboratory, Department of Radiology, Austin Health, Heidelberg, VIC, Australia; 3 Melbourne Medical School, The University of Melbourne, Parkville, VIC, Australia; 4 Department of Endocrinology, Austin Health, Heidelberg, VIC, Australia

**Keywords:** CT brain, deep learning—artificial intelligence, DEXA (Dual energy xray absorptiometry), opportunistic screening opportunistic screening with routine data, osteopenia and osteoporosis

## Abstract

**Objectives:**

CT brain (CTB) scans are frequently performed in older adults, a population at increased risk of osteoporosis and low bone mineral density (BMD), presenting an opportunity for opportunistic screening. This study aims to evaluate an automated deep learning approach for opportunistic screening of low BMD and osteoporosis from routine CTB imaging.

**Materials and methods:**

A single-centre retrospective analysis was conducted on 2,014 patients (mean age 69.7 ± 14.9 years; 61% female) who underwent non-contrast CTB and dual-energy x-ray absorptiometry (DEXA) within one year of each other. A convolutional neural network incorporating automatically selected CTB slices cranial to the lateral ventricles, as well as age and sex, was trained to perform two binary classification tasks: low BMD screening (*T*-score < –1.0) and osteoporosis screening (*T*-score ≤ –2.5). Model performance was evaluated on a 10% hold-out test set using AUC, balanced accuracy, sensitivity, specificity, positive predictive value, and negative predictive value, with subgroup analysis by sex.

**Results:**

22% of patients scanned had normal BMD, 44% had osteopenia, and 34% had osteoporosis. For low BMD screening, the model achieved an AUC of 0.83 (95% CI: 0.76–0.90), with AUCs of 0.88 in females and 0.76 in males. For osteoporosis screening, the model achieved an AUC of 0.78 (95% CI: 0.72–0.85), with AUCs of 0.78 in females and 0.74 in males.

**Conclusion:**

Automated analysis of routine CT brain imaging showed good discriminatory performance for opportunistic low BMD screening, particularly among females. With further validation, this approach could support earlier identification of at-risk individuals using imaging already acquired in routine clinical care.

## Introduction

Osteoporosis is prevalent worldwide and is characterised by decreased bone mineral density (BMD) and deterioration of bone microarchitecture, placing patients at risk of fragility fractures (1, 2). Despite its significant health implications, osteoporosis remains widely underdiagnosed. While osteoporosis is associated with the highest individual fracture risk, a substantial proportion of fragility fractures occur in individuals with osteopenia (3). Low BMD (*T*-score < −1.0), encompassing both osteopenia and osteoporosis, therefore represents a broader at-risk population relevant to screening (3, 4). Low BMD is common, affecting an estimated 67% of Australians aged 50 years and older (4). The downstream economic burden of osteoporosis-related fractures is substantial, estimated at EUR 56.9 billion across the EU/UK/Switzerland and USD 25.3 billion in the United States (4–6). Early identification of low BMD and osteoporosis is therefore important to enable timely intervention to reduce the risk of fragility fractures.

The current standard for assessing bone mineral density relies on dual-energy x-ray absorptiometry (DEXA), with diagnostic thresholds based on *T*-scores (osteoporosis defined as ≤ –2.5, and osteopenia as −2.5 < *T*-score < –1.0 at the femoral neck or lumbar spine), alongside fracture-based diagnosis in the presence of low trauma fractures for osteoporosis. However, at-risk patients often do not undergo DEXA in routine clinical practice, limiting opportunities for early identification (3). This gap has led to growing interest in opportunistic screening approaches using routinely acquired imaging. Computed tomography (CT) scans frequently capture skeletal regions suitable for opportunistic assessment of bone mineral density without additional imaging or radiation exposure. Prior studies have demonstrated the feasibility of this approach in the axial skeleton, using abdominal and thoracic CT to assess vertebral bone density (7, 8). These studies were conducted in patients imaged for abdominal and thoracic indications, however the applicability of such approaches to other skeletal regions captured on routine imaging is less well established.

CT brain (CTB) imaging is frequently performed in older adults presenting to emergency and acute-care settings following a fall or with conditions associated with increased fall risk, such as head injury, delirium, or cerebrovascular events (9–11). As falls are an independent risk factor for fragility fracture (12), the frequent use of CTB in this patient population creates an opportunity to screen for low BMD and osteoporosis opportunistically. Prior CTB-based studies have evaluated manually measured frontal skull attenuation for discriminating DEXA-defined osteoporosis and low BMD. Na et al. reported an AUC of 0.775 for osteoporosis discrimination (13), while subsequent evaluation in a more heterogeneous cohort by Lim et al. reported AUCs of 0.596 for osteoporosis and 0.629 for low BMD (14). These studies suggest that skull-derived CT image features may be informative for low BMD and osteoporosis screening. However, reliance of these approaches on manual measurement of selected skull regions may limit scalability and require additional specialist input, making routine implementation in clinical workflows challenging. In this context, applying deep learning to CTB may enable automated opportunistic screening for low BMD and osteoporosis within existing imaging workflows, facilitating identification of patients who may benefit from further evaluation.

This study evaluates a fully automated SE-ResNet18 deep learning model incorporating routine non-contrast CTB images with age and sex to perform two binary classification tasks: screening for low BMD (*T*-score < –1.0) and osteoporosis (*T*-score ≤ –2.5), using femoral neck DEXA *T*-score as the reference standard. Model performance was assessed on an independent hold-out test set, with subgroup analysis by sex.

## Materials and methods

### Study design

This was a retrospective single centre cohort study. Approval from the Institutional Review Board was obtained and informed consent was not required.

#### Data source

All study subjects were identified using the Radiology Information System (RIS), (Agfa v6.3.3) from Austin Health (Melbourne, Australia).

Images were retrieved in Digital Imaging and Communications in Medicine (DICOM) format and anonymised using the python package DicomAnonymizer (15).

Femoral neck *T*-scores were obtained programmatically with python code from DEXA reports stored in pdf format in our Picture Archiving Communication System (Agfa Enterprise Imaging v 8.2). To validate automated extraction, 150 cases were manually reviewed, including 50 each from the normal BMD (*T*-score ≥ –1.0), osteopenia (–2.5 < *T*-score < –1.0), and osteoporosis (*T*-score ≤ –2.5) groups. Automatically extracted femoral neck *T*-scores matched the original DEXA reports in all 150 cases.

#### Inclusion and exclusion criteria

2051 patients who underwent both DEXA and non-contrast CTB within ±1 year of the DEXA scan between 1st January 2010 and 14th December 2020 were identified. This selection criterion was chosen to maintain reasonable temporal proximity while allowing inclusion of post-DEXA CTBs, as anti-resorptive treatment effects on femoral neck BMD are low within one year (16). If multiple CTB studies were available within the ±1 year timeframe in relation to DEXA, the earliest CTB was selected to reduce the potential influence from interval clinical events.

A portion of the dataset in this study (221 subjects) overlaps with data from a previously published study, which aimed to evaluate the feasibility of opportunistic osteoporosis screening using manually obtained Hounsfield units from the frontal bone on CTB (14). These subjects were included as part of the full cohort and were not handled separately during data partitioning, model training, or test-set evaluation.

**Figure 1:**
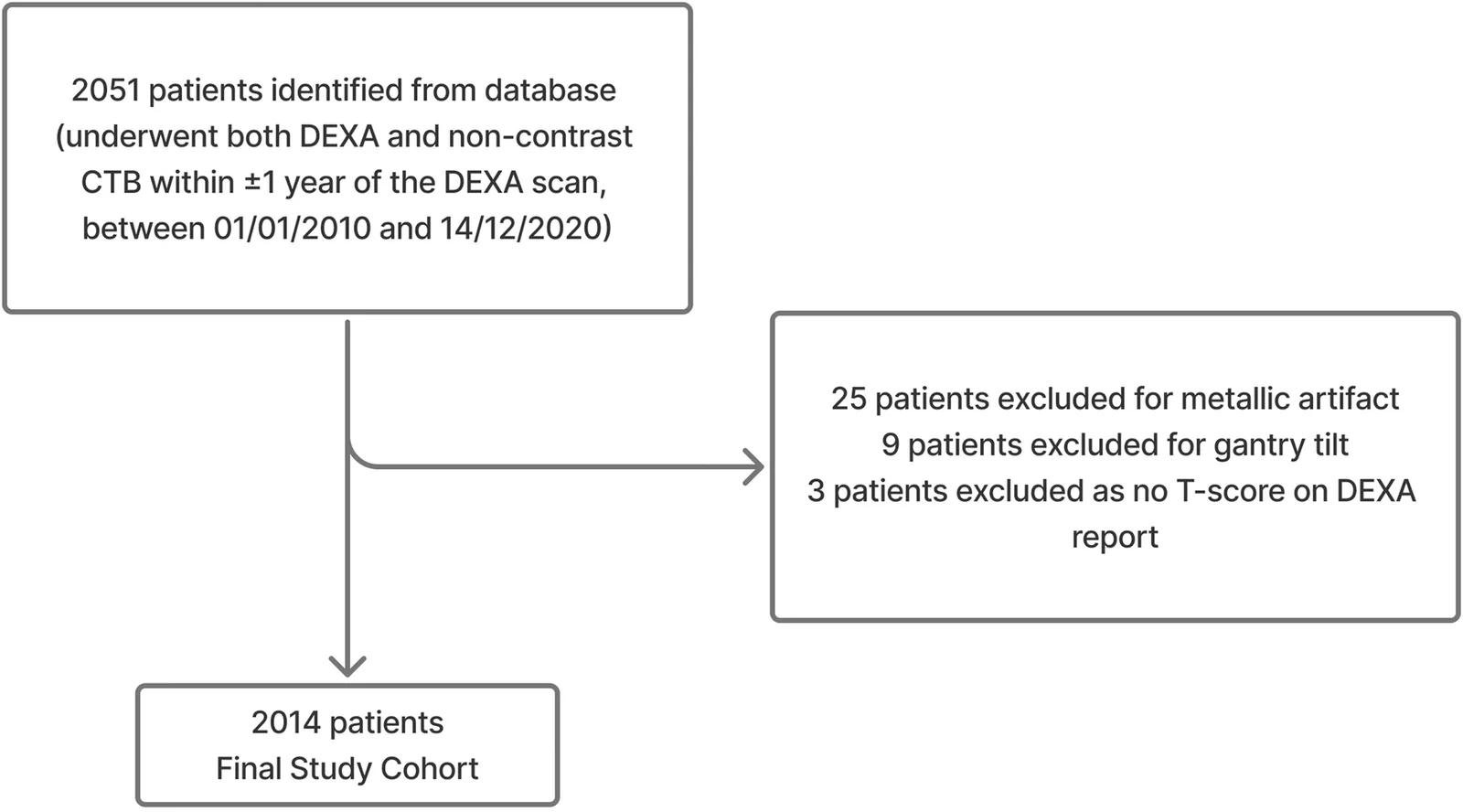
Flow diagram of patient cohort selection.

Subjects with CTBs conducted with gantry tilt and where metallic artifacts significantly impacted image quality and/ or bone visualisation, as reviewed by two experienced radiologists (NK, RPL) in consensus, were removed from the dataset (Figure 1). Patients lacking a valid femoral neck *T*-score on their DEXA reports were also excluded. Mean ± SD slice thickness across the entire dataset was 4.18 ± 0.93 mm.

CTBs were acquired on multiple systems from three vendors, due to equipment replacement over the study period. Detailed scanner models and representative acquisition parameters are provided in Supplementary Materials. CT brain axial slices reconstructed with a soft tissue kernel were used because they were available for all studies. Bone kernel reconstructions were not always generated and were therefore not available for all patients.

#### Data pre-processing

A slice (ideally immediately) cranial to the lateral ventricles was selected as the central reference point for image extraction, consistent with prior studies identifying this level as a stable and informative landmark for skull and frontal bone analysis (13). A standardised slice level was used to improve consistency of skull imaging provided to the model. This level was identified using a publicly available pretrained multi-class U-Net model (17), which segmented the ventricles on each CTB slice. The number of pixels corresponding to the segmented ventricles on each slice was then analysed, the slice with the highest pixel count was taken to represent the mid-lateral ventricles, and the reference slice was selected by progressing cranially until ventricular-system pixels fell below a predefined threshold, as detailed in the Supplementary Materials.

In a random sample of 50 studies reviewed by an experienced radiologist (NK), the algorithm selected the exact target slice in 40 cases (80%). In all cases (100%), the radiologist-defined target slice was contained within the 12-slice extraction window (see Model Input). The mean absolute slice difference was 0.3, median absolute slice difference was 0 slices, with a maximum difference of 3 slices.

Standard data augmentation was performed with the open-source framework MONAI (18) to introduce heterogeneity to the training data, including random rotation, affine shear, elastic deformation, Gaussian noise, and random zoom. Further details are available in Supplementary Materials.

Age was normalised using z-score normalisation. The mean and standard deviation were calculated from the training data only and then applied to the corresponding validation and hold-out test sets. Sex was encoded numerically (i.e., 0 for male, 1 for female).

#### Ground truth

Ground truth for each patient was determined using the femoral neck *T*-score obtained from their DEXA scan. DEXA scans were performed on one of two machines, GE Lunar Prodigy (GE Healthcare Lunar) or Hologic Horizon (Hologic Inc.), with the Hologic system replacing the GE system during the study period. The replacement system underwent manufacturer supplied calibration before clinical use.

Based on the *T*-scores, a binary label was assigned to each patient. Two scenarios were explored where a binary classifier could provide useful insights:

- Low BMD screening (Osteoporosis and Osteopenia)
  If the femoral neck *T*-score is <−1.0, the patient is labelled as low BMD, encompassing both osteopenia (−1.0 > *T*-score > −2.5) and osteoporosis (*T*-score ≤ −2.5). Otherwise, the patient is labelled as normal BMD. Here, the model's output would reflect the likelihood that the patient has low BMD.
- Osteoporotic BMD screening
  If the femoral neck *T*-score is ≤−2.5, the patient is labelled as osteoporotic. Otherwise, the patient is labelled as non-osteoporotic. In this case, the model's output would indicate the likelihood of osteoporosis.

#### Data partitions

A stratified splitting approach was used in this study due to class imbalance within the dataset. This ensured that the training, validation, and test sets maintained similar distributions of patients by label, age, and sex for more reliable and generalisable performance metrics across all folds. From the full dataset of 2,014 samples, 202 (10%) were set aside as a hold-out test set. This hold-out test set was not used for model development and was only used for final performance evaluation. The remaining 1,812 samples were used for 5-fold cross-validation, with each fold using an 80/20 split for training and validation. The same test set and cross-validation splits were applied to both the osteoporosis and low BMD screening scenarios.

#### Model architecture

Three candidate convolutional neural network (CNN) architectures were preliminarily evaluated for classification. These were: ResNet18 (19), DenseNet121 (20), and SE-ResNet18 (21). Model selection was based on mean five-fold cross-validation AUC. SE-ResNet18 achieved the highest AUC for both osteoporosis BMD screening and low BMD screening and was therefore selected as the final architecture. Details of model comparison and performance metrics for all evaluated architectures are provided in Supplementary Materials.

#### Model input

**Figure 2:**
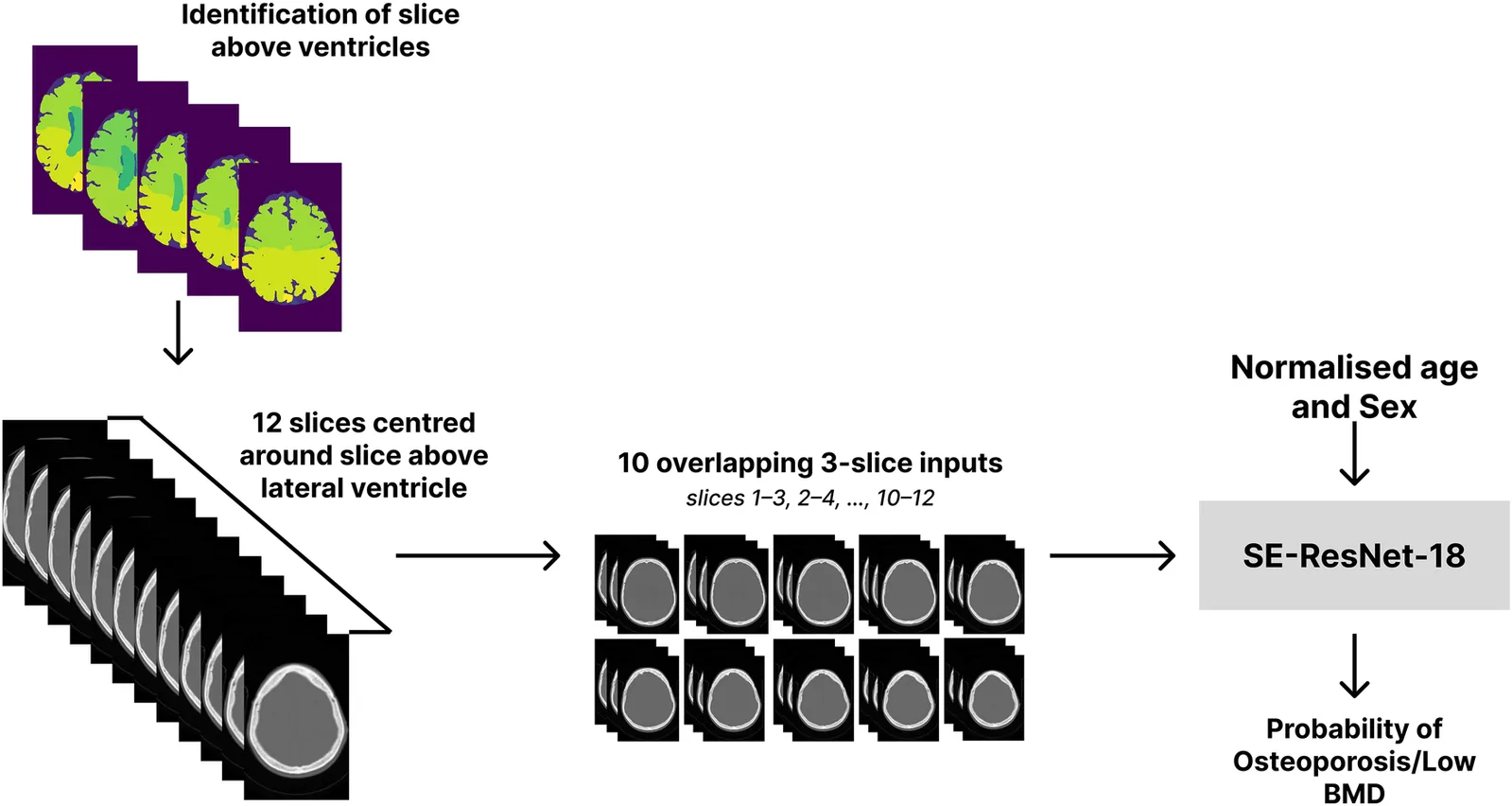
For each patient, 12 adjacent CT brain slices centred on the automatically selected slice above the lateral ventricles were extracted. Ten overlapping three-slice inputs were generated using a sliding-window approach and combined with normalised age and sex for prediction by the SE-ResNet18 model.

To improve model input variety, 12 adjacent axial CT slices centred on the automatically selected reference slice were extracted for each patient. These CTB slices were not resampled to a standardised slice spacing prior to model training. Therefore, the extracted 12-slices may represent different cranio-caudal anatomical coverage depending on the reconstructed slice thickness. Due to the three-channel input structure of CNN architectures, each model input consisted of three consecutive axial slices, with one slice assigned to each input channel. From the 12 extracted slices, 10 overlapping three-slice inputs (slices 1–3, 2–4, …, 10–12) were generated using a sliding-window approach and presented to the model during training and inference (Figure 2).

The model's final fully connected layer was modified to include patient-level demographic data (age and sex) as auxiliary inputs.

#### Oversampling

Oversampling was explored during training to assess whether balancing representation across both labels (0 and 1) and sexes (male and female) would improve model performance. This was implemented using weighted random sampling, where images from less common label–sex groups were more likely to be selected by the training data loader. As oversampling did not lead to improved performance during internal validation, it was not applied in the final model. Further details regarding the oversampling experiments can be found in Supplementary Materials.

#### Model training

The SE-ResNet18 was initialised without pretrained weights and trained using ADAM optimisation over 35 epochs, with a batch size of 128 and weight decay of 1 × 10^−4^. The 35-epoch upper limit was selected based on preliminary training experiments showing that validation AUC generally plateaued before epoch 30. For each fold, the checkpoint achieving the highest validation AUC was retained. Learning rate was initialised at 3 × 10^−3^, a learning rate scheduler (ReduceLROnPlateau) was utilised with factor of 0.1 and patience of 2. Binary Cross Entropy Loss function was used to calculate loss. Training was performed on an Nvidia RTX 3090 with 24Gb of VRAM, using Python 3.10.9 and Pytorch 1.13.1. Details of other packages are provided in Supplementary Materials.

The model's output was a probability between 0 and 1 for both scenarios.

### Final model selection

For final model selection, the checkpoint with the highest validation AUC from each cross-validation fold was compared, and the fold-specific model with the highest validation AUC was then selected for evaluation on the independent hold-out test set. This model was not retrained on the full development cohort or ensembled with the other fold-specific models.

#### Decision threshold of test set

To convert model outputs (probabilities between 0 and 1) into binary classifications, decision thresholds were derived from the validation predictions of the fold corresponding to the selected final model and applied without further adjustment to the independent hold-out test set.

Given the intended application as a screening tool, thresholds were selected to prioritise sensitivity. Specifically, the highest threshold to maintain a minimum 80% sensitivity on the validation set was selected for both the low BMD and osteoporosis screening tasks. This sensitivity-target threshold was chosen with the aim of reducing missed cases while limiting false-positive classifications and unnecessary downstream DEXA referrals. Test-set results were then reported using these validation set derived thresholds. Thresholds derived using Youden's J statistic (maximising the sum of sensitivity and specificity) were also evaluated for reference (22, 23).

#### Inference

For inference on the test set, 12 unique adjacent axial slices centred on the algorithm-selected slice cranial to the lateral ventricles were extracted for each patient. These 12 slices were used to generate 10 overlapping three-slice inputs using a sliding window approach (Figure 2). The model generated a probability for each of the 10 inputs, and the final patient-level prediction was obtained by averaging these 10 probabilities.

#### Model evaluation

Model performance was assessed using balanced accuracy, sensitivity, specificity, positive predictive value (PPV), negative predictive value (NPV), and the area under the receiver operating characteristic curve (AUC). Balanced accuracy, defined as the weighted average of class-wise accuracies, was chosen to account for potential class imbalance. Sensitivity and specificity were used to evaluate the model's ability to correctly identify positive and negative cases respectively. Among these metrics, AUC was prioritised due to its threshold-independent nature and its ability to capture the model's performance across the full spectrum of decision thresholds. This makes it suitable for evaluating classifier behaviour where threshold selection may vary. Subset analysis was performed based on sex to assess for model performance variation across male and female subgroups. All statistical analyses were performed using Python (version 3.10.9) with full details provided in Supplementary Materials.

#### Comparison with logistic regression model

Given the final model (SE-ResNet18) takes imaging, age and sex as inputs, we also assessed whether imaging provides additional predictive performance beyond prediction using only age and sex. Therefore, a baseline logistic regression model was trained using only age and sex. Delong's test (24) was used to determine whether the difference in AUC between the SE-ResNet18 model and the logistic regression baseline was statistically significant, a *p*-value of less than 0.05 was considered statistically significant.

#### Visualisations

Saliency maps were employed to aid interpretability. Occlusion-based attribution was applied using a sliding window of size 15 × 15 pixels, with a stride of 8 pixels, enabling localised analysis of feature importance across the input image.

Occlusion attribution maps were stratified by age group (<65 vs. ≥65 years) and sex (male vs. female) for qualitative visual assessment. An age cutoff of 65 years was used to align with commonly used clinical thresholds for older adult populations (25, 26). Attribution visualisations were generated by averaging occlusion attributions across the middle slice (slice above the lateral ventricle) of each test series within each respective subgroup. These attribution maps were intended to assist in qualitative assessment of model behaviour across subgroups, no formal statistical comparison of attribution patterns was performed.

## Results

Of 2,051 initially identified patients, 37 were excluded (acquisition with gantry tilt, *n* = 9; and metallic artefacts impacting bone visualisation *n* = 25, absence of *T*-score from report, *n* = 3). A total of 2014 patients (mean age ± SD: 69.7 ± 14.9 years; 61% female) were included for training and testing (Figure 1) with the full and hold-out test cohort characteristics presented in Tables 1, 2 respectively.

**Table 1:** Properties of full dataset by BMD group (osteoporosis, osteopenia, normal), with age, *T*-score, and subgroup counts stratified by sex and age. %F/%Female Subgroup indicates the proportion within female subgroup of full dataset, %M/%Male Subgroup indicates the proportion within male subgroup of full dataset. The far-right column shows each diagnostic group as a percentage of the full dataset.

BMD group	Age		*T*-Score		Sex						N (% Full Dataset)
	Mean	Std	Mean	Std	Female			Male			
					Age < 65 (%F)	Age ≥ 65 (%F)	Total (% Female Subgroup/%Full Dataset)	Age < 65 (%M)	Age ≥ 65 (%M)	Total (% Male Subgroup/ %Full Dataset)	
Osteoporosis *T*-score ≤ −2.5	75.99	12.36	−3.24	0.67	71 (6%)	412 (33%)	483 (39%/24%)	42 (5%)	155 (20%)	197 (25%/10%)	680 (34%)
Osteopenia −2.5 < *T*-score < −1.0	68.77	14.25	−1.79	0.40	173 (14%)	366 (30%)	539 (44%/27%)	137 (18%)	214 (27%)	351 (45%/17%)	935 (44%)
Normal *T*-score ≥ −1.0	61.74	15.35	−0.29	0.65	125 (10%)	88 (7%)	213 (17%/11%)	117 (15%)	114 (15%)	231 (30%/11%)	399 (22%)
Total	69.66	14.86	−1.95	1.22	369 (30%)	866 (70%)	1,235 (100%/61%)	296 (38%)	483 (62%)	779 (100%/39%)	2,014

**Table 2:** Properties of test dataset by BMD group (osteoporosis, osteopenia, normal), with age, *T*-score, and subgroup counts stratified by sex and age. %F/%Female Subgroup indicates the proportion within female subgroup of test dataset, %M/%Male Subgroup indicates the proportion within male subgroup of test dataset. The far-right column shows each diagnostic group as a percentage of the test dataset.

BMD group	Age		*T*-Score		Sex						N (% Test Set)
	Mean	Std	Mean	Std	Female			Male			
					Age < 65 (%F)	Age ≥ 65 (%F)	Total (% Female Subgroup/ %Test Set)	Age < 65 (%M)	Age ≥ 65 (%M)	Total (% Male Subgroup/ %Test Set)	
Osteoporosis *T*-score ≤ −2.5	75.43	13.25	−3.30	0.70	5 (4%)	43 (35%)	48 (39%/24%)	5 (6%)	15 (19%)	20 (26%/10%)	68 (34%)
Osteopenia −2.5 < *T*-score < −1.0	68.28	13.72	−1.76	0.39	20 (16%)	39 (31%)	59 (48%/29%)	16 (21%)	22 (28%)	38 (49%/19%)	97 (48%)
Normal *T*-score ≥ −1.0	61.95	14.69	−0.35	0.60	12 (10%)	5 (4%)	17 (14%/8%)	11 (14%)	9 (12%)	20 (26%/10%)	37 (18%)
Total	69.52	14.50	−2.02	1.18	37 (30%)	87 (70%)	124 (100%/61%)	32 (41%)	46 (59%)	78 (100%/39%)	202 (100%)

### Low BMD screening

The SE-ResNet–18 model achieved an AUC of 0.83 (95% CI: 0.76–0.90) on the hold-out test set. A decision threshold of 0.66, calculated from the validation set to target 80% sensitivity, resulted in balanced accuracy of 0.75, sensitivity of 0.77, specificity of 0.68, PPV of 0.91, and NPV of 0.40 on the test set.

**Figure 3:**
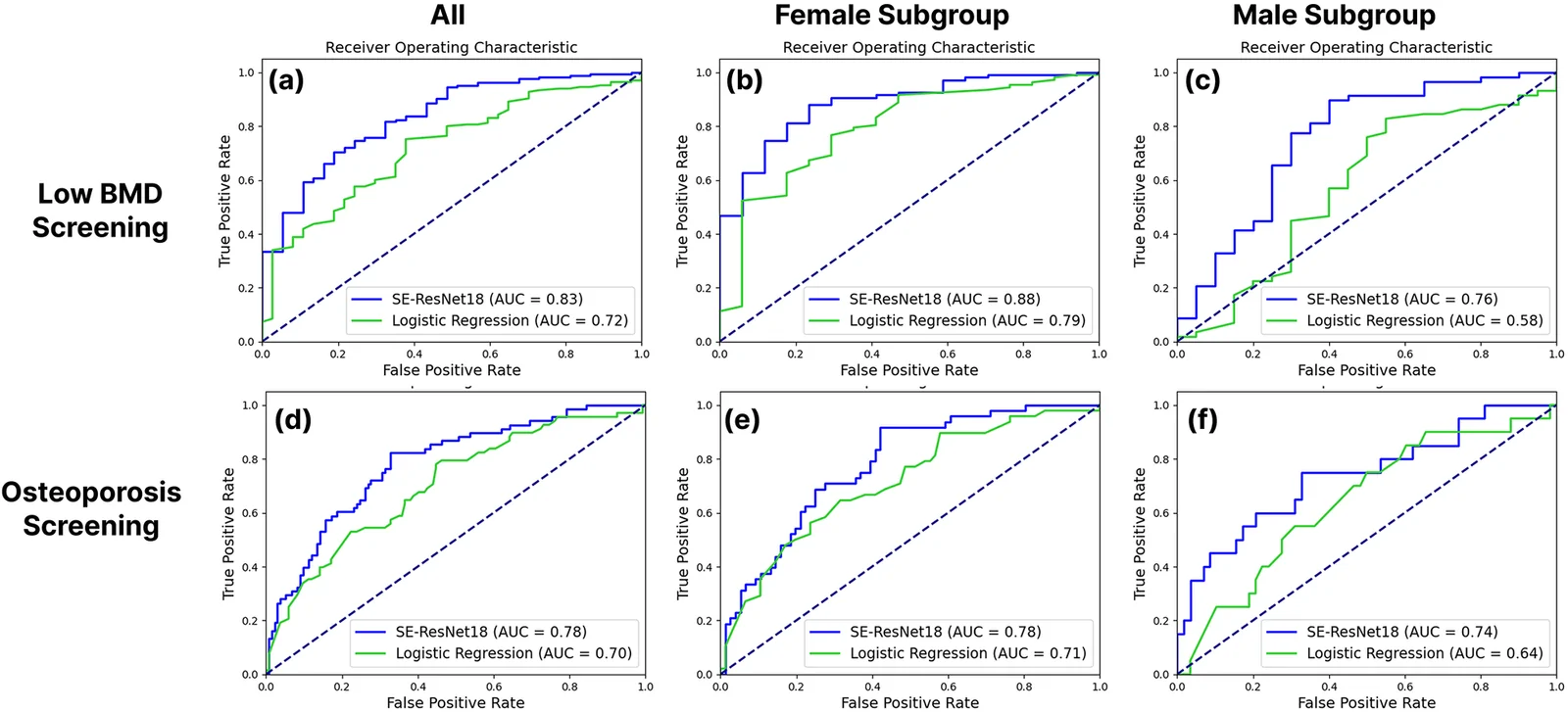
Receiver operating characteristic (ROC) curves. Low BMD Screening (*T* score < −1.0) for **(a)** all subjects, **(b)** female subgroup, and **(c)** male subgroup. Osteoporosis BMD Screening (*T* score ≤ −2.5) for **(d)** all subjects, **(e)** female subgroup, and **(f)** male subgroup.

Subgroup analysis demonstrated higher performance in females, with an AUC of 0.88, balanced accuracy of 0.86, and a high sensitivity of 0.91 at the expense of specificity (0.59). In males, performance was lower, with an AUC of 0.76, balanced accuracy of 0.58, sensitivity of 0.52 and specificity of 0.75 (Figure 3, Table 3). Performance using the Youden index-derived threshold is presented in Table 3 for comparison.

**Table 3:** SE-ResNet18 performance of Low BMD screening scenario (*T*-score < −1.0) on the hold out test set, including subgroup analysis by sex. AUC was compared between SE-ResNet18 and logistic regression to assess whether imaging inputs added predictive value beyond age and sex alone. Statistically significant improvements in AUC are highlighted in bold. S, sensitivity-prioritised threshold targeting 80% sensitivity on the validation set; Y, Youden index-derived threshold maximising sensitivity + specificity on the validation set.

Subset	Sample size	SE-ResNet18 AUC	SE-ResNet18 AUC 95% CI (Delong)	Logistic regression AUC	*p*-value (Delong)	Threshold strategy	Threshold	Balanced accuracy	Sensitivity	Specificity	PPV	NPV
Overall	202	0.83	0.76–0.90	0.72	**0** **.** **004**	S	0.66	0.75	0.77	0.68	0.91	0.40
						Y	0.75	0.67	0.63	0.84	0.95	0.34
Female	124	0.88	0.81–0.96	0.79	**0**.**049**	S	0.66	0.86	0.91	0.59	0.93	0.50
						Y	0.75	0.79	0.79	0.82	0.97	0.38
Male	78	0.76	0.62–0.89	0.58	**0**.**047**	S	0.66	0.58	0.52	0.75	0.86	0.35
						Y	0.75	0.47	0.34	0.85	0.87	0.31

Compared with the logistic regression baseline trained on age and sex alone, the SE-ResNet–18 model demonstrated statistically significant AUC improvements in the overall cohort (0.83 vs. 0.72, *p* = 0.004), females (0.88 vs. 0.79, *p* = 0.049), and males (0.76 vs. 0.58, *p* = 0.047; Table 3).

### Osteoporosis BMD screening

The SE-ResNet–18 model achieved an AUC of 0.78 (95% CI: 0.72–0.85) on the hold-out test set. A decision threshold of 0.21, derived from the validation set to target 80% sensitivity, resulted in a balanced accuracy of 0.71, with sensitivity of 0.79, specificity of 0.67, PPV of 0.55, and NPV of 0.87 on the test set.

Subgroup analysis for females demonstrated an AUC of 0.78, balanced accuracy of 0.69, sensitivity of 0.88, and specificity of 0.58. In the male subgroup, AUC was 0.74, balanced accuracy was 0.74, sensitivity was 0.60, and specificity was 0.79 (Figure 3, Table 4). Performance using the Youden index-derived threshold is reported in Table 4 for comparison.

**Table 4:** SE-ResNet18 performance of osteoporosis BMD screening scenario (*T*-score ≤ −2.5) on the hold out test set, including subgroup analysis by sex. AUC was compared between SE-ResNet18 and logistic regression to assess whether imaging inputs added predictive value beyond age and sex alone. Statistically significant improvements in AUC are highlighted in bold. S, sensitivity-prioritised threshold targeting 80% sensitivity on the validation set; Y, Youden index-derived threshold maximising sensitivity + specificity on the validation set.

Subset	Sample size	SE-ResNet18 AUC	SE-ResNet18 AUC 95% CI (Delong)	Logistic regression AUC	*p*-value (Delong)	Threshold strategy	Threshold	Balanced accuracy	Sensitivity	Specificity	PPV	NPV
Overall	202	0.78	0.72–0.85	0.70	**0** **.** **001**	S	0.21	0.71	0.79	0.67	0.55	0.87
						Y	0.35	0.74	0.59	0.81	0.62	0.80
Female	124	0.78	0.70–0.86	0.71	**0**.**048**	S	0.21	0.69	0.88	0.58	0.57	0.88
						Y	0.35	0.70	0.71	0.70	0.60	0.79
Male	78	0.74	0.60–0.87	0.64	0.066	S	0.21	0.74	0.60	0.79	0.50	0.85
						Y	0.35	0.79	0.30	0.97	0.75	0.80

The SE-ResNet–18 model outperformed the baseline logistic regression in the overall cohort (AUC: 0.78 vs. 0.70, *p* = 0.001) and in females (AUC: 0.78 vs. 0.71, *p* = 0.048). Lower AUC observed in males did not reach statistical significance (AUC: 0.74 vs. 0.64, *p* = 0.066). (Table 4).

### Saliency map visualisation

Occlusion visualisation showed that the skull had the greatest influence on model predictions in both scenarios. When comparing the two scenarios, the skull exhibited stronger overall attribution in the low BMD screening scenario than in the osteoporosis BMD screening. In the low BMD scenario, attributions were noted to focus on specific skull regions, particularly the occipital and frontal bones. In contrast, the osteoporosis BMD screening scenario showed more diffuse attention across the entire skull, with no distinct focus on any specific skull region.

**Figure 4:**
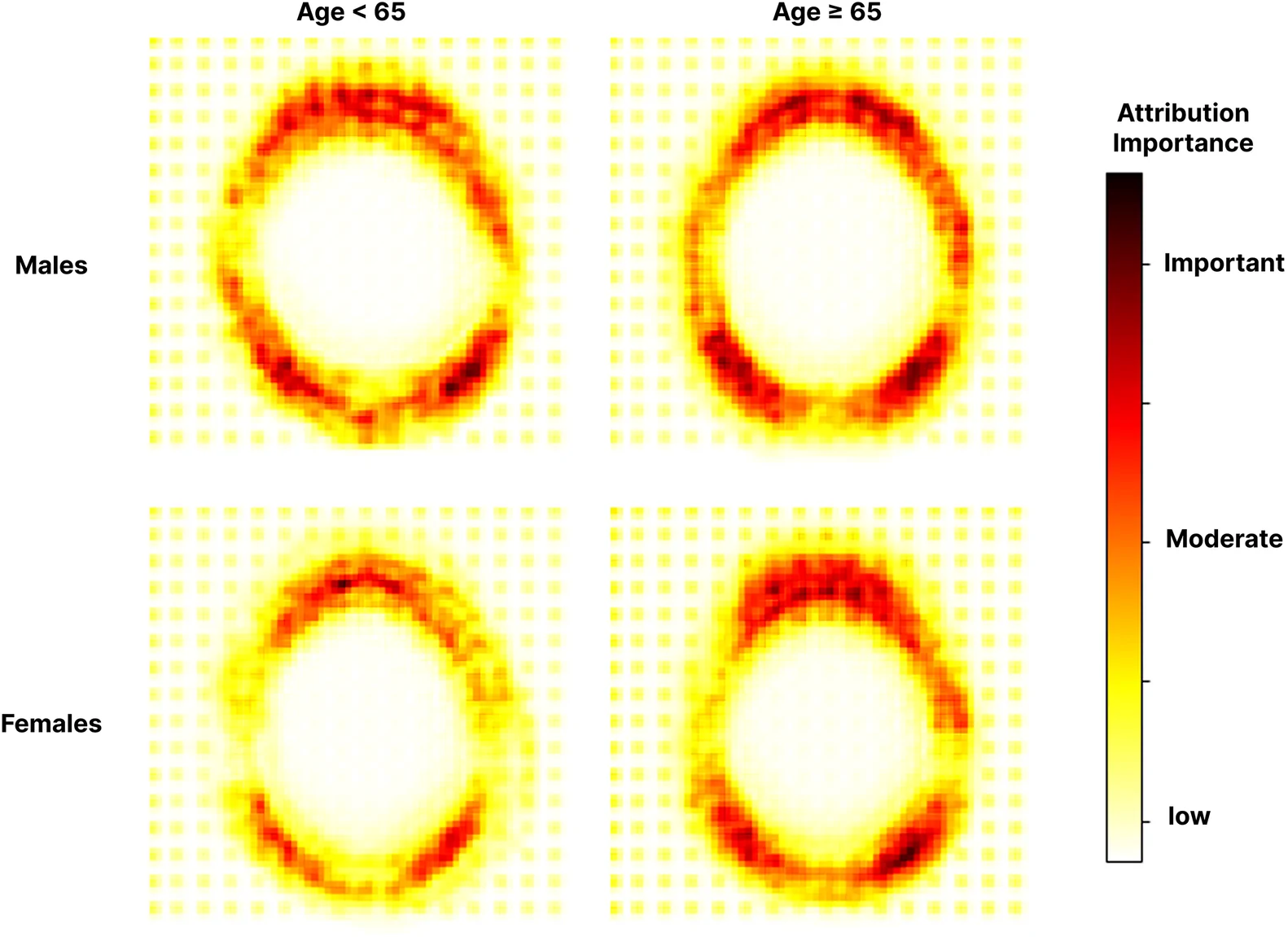
Low BMD screening—average occlusion attribution, stratified by sex and age group. Four maps show the average attributions across four patient subgroups: male < 65, male ≥ 65, female < 65, and female ≥ 65. Values represent the average patient attributions within each group.

**Figure 5:**
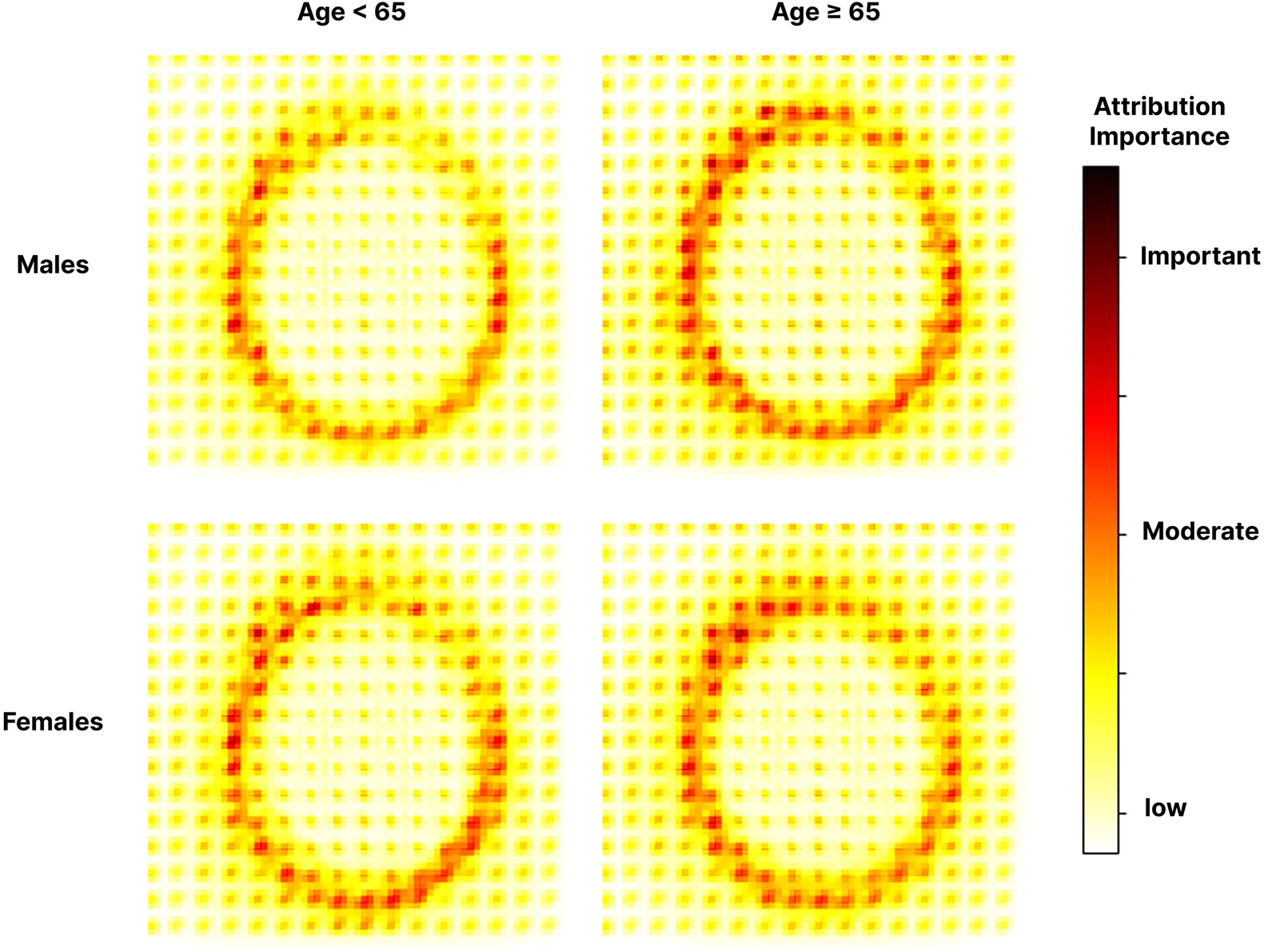
Osteoporosis BMD screening—average occlusion attribution, stratified by sex and age group. Four maps show the average attributions across four patient subgroups: male < 65, male ≥ 65, female < 65, and female ≥ 65. Values represent the average patient attributions within each group.

On qualitative visual assessment, attribution patterns appeared broadly similar between males and females and between patients aged <65 and ≥65 years (Figures 4, 5).

Our model is made available online for the scientific community at https://github.com/AH-AI-in-Radiology/CTB_Osteoporosis_Screening.

## Discussion

This study demonstrates the feasibility of using routine CT brain imaging for automated opportunistic screening of low BMD. The model achieved promising discriminatory performance in the low BMD screening task (AUC: 0.83), particularly among females (AUC: 0.88). This supports its potential role as a triage tool to identify patients who may benefit from further DEXA evaluation within current clinical workflows. Performance in the osteoporosis screening task was more modest (AUC: 0.78). One possible explanation for the stronger performance in the low BMD screening task is that morphological changes in cranial bone structure may be more visually distinct between normal bone density (*T*-score ≥ −1.0) and any level of demineralisation (*T*-score < −1.0), whereas differences between osteopenia and osteoporosis may be more subtle. The capability to screen for low bone density in women is important because most of the fragility fracture burden occurs in women with osteopenia rather than established osteoporosis (3).

CT brain-based approaches for opportunistic osteoporosis screening have previously relied on manually measured frontal bone attenuation and have demonstrated variable performance across studies (13, 14). The present study evaluated an automated deep learning approach incorporating spatial imaging features and demographic information, with performance assessed on a hold-out test set. This approach extends prior work by moving beyond manual attenuation measurements and supports the potential for automated opportunistic screening using CTB.

Sex-specific differences in model performance were also observed, with better performance in females. This disparity was especially pronounced for low BMD screening, where the model achieved an AUC of 0.88 in females compared to 0.76 in males. Several factors may contribute to this difference. First, *T*-scores in males are derived from female reference populations rather than male-specific cohorts, which may weaken the correlation between skull measurements and fracture risk in men. Second, biological and dataset-related factors likely contribute, including the higher prevalence of osteoporosis in women and the more consistent radiological bone patterns observed in female patients (27). Given that older women are at greater risk of osteoporosis-related fragility fractures due to accelerated post-menopausal bone-loss, early identification in this population is particularly critical (28). The comparatively lower AUC observed in males suggests that the current model may have more limited applicability in this subgroup. The lower sensitivity (0.52) observed in males at the selected threshold also suggests that this threshold may be less suitable for male patients, and that subgroup-specific threshold calibration may be required.

Comparison with a logistic regression baseline confirmed the added predictive ability of imaging for screening purposes, particularly in females. In males however, model performance did not significantly exceed the logistic regression baseline in the osteoporosis screening scenario. This may reflect insufficient statistical power but also sex-related differences of imaging features. Specifically, the features used to infer osteoporosis—such as trabecular bone patterns and radiodensity characteristics—may be less pronounced or more variable in males (27).

Visualisations showed that the model primarily focused on the bony calvarium when making predictions. The strong attribution to the frontal and occipital regions in the low BMD screening scenario may reflect frontal and occipital cortical bone thickness as a surrogate for skeletal health (27). In the osteoporosis BMD screening scenario, the attribution was more diffuse. This may be attributed to confounding from conditions such as hyperostosis frontalis interna, which is prevalent in females aged over 65 (29) and can cause frontal bone thickening.

Limitations of this study include the dataset's origin from a single institution serving a metropolitan, ethnically diverse catchment area, as well as its skew toward female participants. These factors may limit the generalisability to other locations and populations, and formal external validation on an independent cohort was not performed. At present, publicly available datasets pairing non-contrast CT brain imaging with DEXA measurements do not exist. CT brain imaging in this study was acquired over a ten-year period across multiple scanner models from three vendors, in routine clinical imaging contexts. In addition, images that were reconstructed at various slice thicknesses (such as 3 mm and 5 mm slices) were not resampled to a standardised slice spacing. Although this acquisition-related heterogeneity reflects routine clinical practice and increases model exposure to scanner and reconstruction variation, all data were derived from a single institution and clinical environment, and external validation remains necessary.

The study cohort was also limited to patients who underwent both CT brain imaging and DEXA, resulting in a higher proportion of osteopenia and osteoporosis that may not reflect the general population. The resulting high prevalence of low BMD may have contributed to the low NPV observed in the low BMD screening task. When multiple eligible CTB examinations were available, the earliest CTB examination was selected instead of the CTB closest to the DEXA. This may have introduced some temporal variability between CTB and DEXA examinations. Use of femoral neck *T*-score alone as the reference standard may also underestimate low BMD or osteoporosis prevalence, as diagnostic classification can be based on the lowest *T*-score across the lumbar spine, total hip, or femoral neck. The small number of normal BMD cases, particularly among females in the hold-out test set, may reduce the precision of threshold-dependent metrics such as specificity and NPV. Accordingly, AUC was prioritised as the primary threshold-independent performance metric. Clinical risk factors such as body mass index (BMI), ethnicity, prior fractures, and medications were not included into model training due to limited availability. We used axial imaging with standard soft tissue reconstruction kernel rather than dedicated bone reconstruction kernel, as the latter was not available in all studies, however this is potentially advantageous for opportunistic screening purposes, as it reflects images routinely acquired in clinical practice.

We also used automated rather than manual identification of the slice above the lateral ventricles. This approach enables a scalable and automated pipeline with minimal user input which we share with the scientific community. As detailed in Methods, validation of a random sample of 50 studies by an experienced radiologist showed that deviations from the radiologist-defined target slice were small, and the target slice was contained within the 12-slice model extraction window in all reviewed cases.

Finally, decision thresholds were selected to prioritise sensitivity for the intended screening application, recalibration is required before use in populations with different disease prevalence or clinical priorities.

We have demonstrated that a deep learning approach applied to routine CT brain images may support opportunistic low BMD screening, particularly in females. Future work incorporating multicentre input data for training may further improve model performance and generalisability, as could integrating additional clinical factors. With appropriate external and prospective validation, such an automated approach could be integrated into clinical workflows to support earlier identification and referral of at-risk individuals, with the potential to improve downstream clinical outcomes and reduce fracture-related morbidity.

## Data Availability

The datasets presented in this article are not readily available because of institutional and ethical restrictions but are available from the corresponding author on reasonable request, subject to approval by the Austin Health Human Research Ethics Committee. Requests to access the datasets should be directed to Stefan.KACHEL@austin.org.au.
