# Supplementary material for: Automated opportunistic screening for low bone mineral density using routine CT brain imaging
Source: Front Radiol. 2026 Aug 14;6:1901958. doi: 10.3389/fradi.2026.1901958 (PMC13522152; doi:10.3389/fradi.2026.1901958)
Supplement: Supplementary file 1 [file Datasheet1.pdf]

## Supplementary materials

### Scanner Details

CT Brain images were acquired on multiple systems from three vendors due to equipment replacement during the study period over time.

| CT Scanner Manufacturer | CT Scanner Model | N |
|---|---|---|
| GE MEDICAL SYSTEMS (Illinois, United States) | LightSpeed VCT | 577 |
| | Revolution CT | 184 |
| | Revolution EVO | 644 |
| SIEMENS (Erlangen, Germany) | SOMATOM Force | 269 |
| | SOMATOM Perspective | 98 |
| TOSHIBA (Tochigi, Japan) | Aquilion | 33 |
| | Aquilion PRIME | 209 |
| Total | | 2014 |

### Scanner Acquisition parameters

Representative acquisition parameters for non-contrast CT brain performed.

| Parameters | Brain |
|---|---|
| Scan Mode | Helical |
| Collimation (mm) | 19.2 |
| Recon thickness ST (mm) | 3 or 5 |
| Rotation time (s) | 1 |
| Pitch | 0.55 |
| kVp | 120 |
| Tube current (mA) | 104 - 214 |

**Data Pre-Processing: Selection of slice above lateral ventricles**

A publicly available pretrained multi-class U-Net model (1) performed semantic segmentation of each CTB slice, labelling each pixel on a slice as a brain structure or "not brain." Pixels labelled as "ventricular system" were counted for each slice, with the slice containing the highest count assumed to represent the middle of the lateral ventricles.

The slice above the ventricles was identified by analysing slices cranial to this level, until the "ventricular system" pixel count dropped below 5% of the maximum, accounting for residual mislabelling (small numbers of pixels which were incorrectly classified as part of the ventricular system due to segmentation inaccuracies). A limit of one-fifth of the scan length was also imposed, beyond which the slice was assumed to lie well cranial to the lateral ventricles.

In a random sample of 50 studies reviewed by an experienced radiologist (NK), the algorithm selected the exact target slice in 40 cases (80%). In all cases (100%), the radiologist-defined target slice was contained within the 12-slice extraction window (see Methods - Model Input). The mean absolute slice difference was 0.3, median absolute slice difference was 0 slices, with a maximum difference of 3 slices.

**Data augmentation**

Augmentations which affect intensity or blur the input images, such as AdjustContrast or GaussianSmooth, were avoided, as there was a concern this would impact the reliable assessment of bone architecture and BMD. Preliminary training also showed that windowing (such as bone windowing) did not improve validation performance, hence the final model did not use windowing.

Data Augmentation Parameters:

RandRotate: range_x=1.57, range_y=1.57

Rand2DElastic: pacing=500, magnitude_range=(0, 50)

RandAffine: shear_range=(0, 1)

RandGaussianNoise: mean=0.0, std=0.1

RandZoom: 0.9 to 1.2

**Model Selection**

Three candidate architectures—ResNet18(2), DenseNet121(3), and SE-ResNet18(4)—were evaluated using 5-fold cross validation. SE-ResNet18 delivered marginally better performance and was ultimately selected. This was likely due to SE-ResNet architecture's incorporation of squeeze-and-excitation (SE) blocks, which enhance channel-wise feature recalibration and contribute to improved classification accuracy (4).

| 5-Fold Cross Validation AUC | Osteoporosis BMD Screening | Low BMD Screening |
|---|---|---|
| DenseNet121 | 0.798 | 0.813 |
| ResNet18 | 0.799 | 0.804 |
| SE-ResNet18 | 0.802 | 0.815 |

**Assessment of oversampling performance**

Oversampling was explored to assess whether balancing representation across both labels (0 and 1) and sexes (male and female) would improve model performance.

In both the osteoporosis and low BMD screening scenarios, oversampling did not improve performance. The five-fold average AUC for osteoporosis screening was 0.802 without oversampling and 0.798 with oversampling. for low BMD screening, it remained 0.815 regardless of oversampling. Therefore, the final models were trained without oversampling.

| 5-Fold Cross Validation AUC SE-ResNet18 | Osteoporosis BMD Screening | Low BMD Screening |
|---|---|---|
| No Oversampling | 0.802 | 0.815 |
| With Oversampling | 0.798 | 0.815 |

**Confusion matrices for threshold-dependent performance**

Hold-out test set confusion matrices are provided for the 80% sensitivity-target thresholds for both screening tasks. Thresholds were selected on the validation set to target approximately 80% sensitivity and then applied to the hold-out test set. These matrices correspond to the operating characteristics reported in Tables 2 and 3 of the main manuscript.

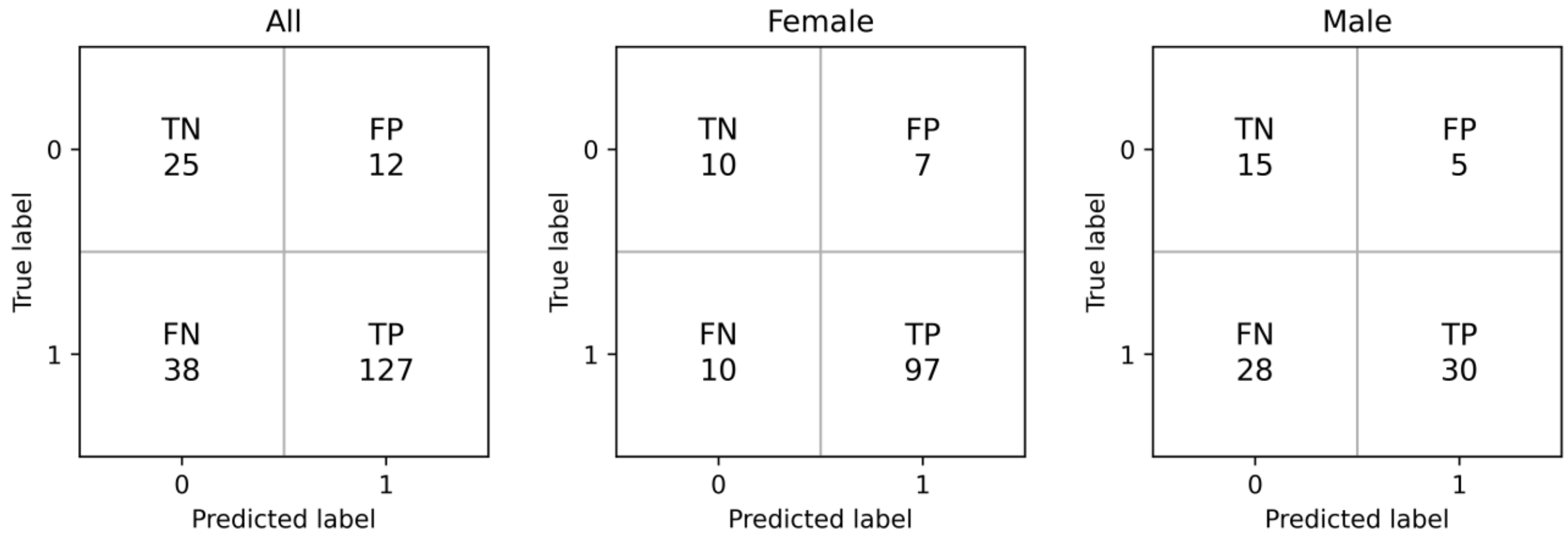


Supplementary Figure 1. Confusion matrices for low BMD screening using the validation-derived 80% sensitivity-target threshold.

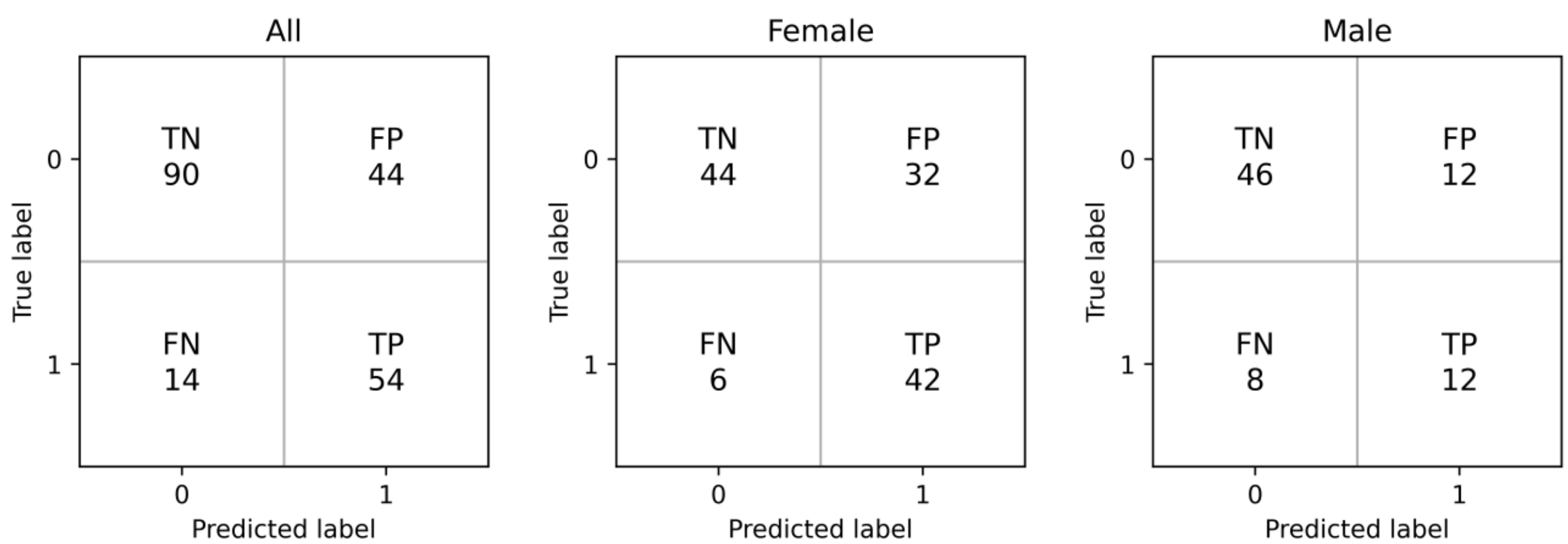


Supplementary Figure 2. Confusion matrices for osteoporosis screening using the validation-derived 80% sensitivity-target threshold.

**Statistical Package Details**

Sensitivity, specificity, positive predictive value (PPV), and negative predictive value (NPV) were computed using direct Python implementations, given their straightforward formulations.

Balanced accuracy (weighted average of class-wise accuracies) and the area under the ROC curve (AUC) were calculated using the sklearn.metrics module from the scikit-learn library.

The DeLong test for comparing AUCs was performed using the MLstatkit Python package, which provides a fast implementation of DeLong's algorithm as described by Sun & Xu (2014) and DeLong et al. (1988) (5, 6).

**List of key python packages**

python 3.10.9
notebook 6.5.2 (Jupyter Notebook for code development)
torch 1.13.1 (Models)
timm 0.6.13 (Python Image Models)
monai 1.1.0 (Image preprocessing and augmentation)
wandb 0.16.3 (Model training logger)
numpy 1.23.4 (Calculations)
pandas 1.5.3 (Data wrangling)
Scikit-learn 1.2.2 (Logistic Regression and Results calculations)

Model and demo code available at: https://github.com/AH-AI-in-Radiology/CTB_Osteoporosis_Screening

## References


1. Cai JC, Akkus Z, Philbrick KA, Boonrod A, Hoodeshenas S, Weston AD, et al. Fully automated segmentation of head CT neuroanatomy using deep learning. Radiology: Artificial Intelligence. 2020;2(5):e190183.
2. He K, Zhang X, Ren S, Sun J, editors. Deep residual learning for image recognition. Proceedings of the IEEE conference on computer vision and pattern recognition; 2016.
3. Huang G, Liu Z, Van Der Maaten L, Weinberger KQ, editors. Densely connected convolutional networks. Proceedings of the IEEE conference on computer vision and pattern recognition; 2017.
4. Hu J, Shen L, Sun G, editors. Squeeze-and-excitation networks. Proceedings of the IEEE conference on computer vision and pattern recognition; 2018.
5. DeLong ER, DeLong DM, Clarke-Pearson DL. Comparing the areas under two or more correlated receiver operating characteristic curves: a nonparametric approach. Biometrics. 1988:837-45.
6. Sun X, Xu W. Fast implementation of DeLong's algorithm for comparing the areas under correlated receiver operating characteristic curves. IEEE Signal Processing Letters. 2014;21(11):1389-93.
